# Home-based physical activity and well-being during infectious diseases: a structural equation modeling study

**DOI:** 10.3389/fpsyg.2025.1732564

**Published:** 2026-01-13

**Authors:** Xiang Peng, Zulkaif Ahmed Saqib, Rashid Menhas

**Affiliations:** 1 College of Physical Education, Hunan City University, Yiyang, China; 2 College of Environmental and Civil Engineering, Chengdu University of Technology, Chengdu, China; 3 Department of Nursing, Shandong Xiehe University, Jinan, China

**Keywords:** home-based physical activity, mental well-being, physical health, preventive measures, respiratory infectious disease

## Abstract

**Background:**

Respiratory infectious diseases that require home isolation and preventive measures seriously disrupt human well-being. Home isolation keeps individuals who do not need hospitalization at home for activities and treatments. Promoting accessible interventions, such as home-based physical activity, is critical for sustaining health during periods of limited mobility. This study helps us understand how home-based activities contribute to building resilience in situations with limited access to traditional health care.

**Objective:**

This study assessed the impact of home-based physical activity on quality of life, psychological resilience, physical and mental health, and sleep quality during respiratory infectious diseases.

**Methods:**

A testable structural model based on social cognitive theory was used to examine the hypotheses of this study. Data were collected using an adapted questionnaire via a web-based survey of 2,200 adults. A convenience sampling approach was used to determine the sample size of this study. Data analysis was performed using structural equation modeling (SEM) with SmartPLS 4.0 to test the hypotheses.

**Results:**

Statistical analysis revealed that infectious diseases significantly impair mental well-being (*β* = −0.241), sleep quality (*β* = −0.092), physical health (*β* = −0.232), and psychological resilience (*β* = −0.276). Home-based physical activity mediates these effects by improving mental health, sleep, and physical health. Significant (green paths) and insignificant (red paths) mediating effects were identified in the research framework and path diagrams. Reliability and validity metrics confirmed the model’s robustness and the distinctness of the constructs.

**Conclusion:**

This study demonstrates that engaging in home-based physical activity enhances psychological resilience, physical health, and sleep quality during infectious disease episodes. Using social cognitive principles, this intervention provides a practical, non-pharmacological approach to combat declining well-being during isolation.

## 1 Introduction

Various diseases affect families and individuals, including their finances, security, employment, and housing (Furushima et al., 2018; Winkler et al., 2005). Worry, tension, anxiety, and other emotions are natural during uncertainty or for those with mental illnesses (Cohen et al., 2009; Wolkoff et al., 2021). Fear, traumatic stress, and anxiety often arise in chronic diseases (Singhal et al., 2024). Many studies have shown that psychological problems are common and have explored their causes (Cohen et al., 2008, 2009; Singhal et al., 2024; Wolkoff et al., 2021). Past research on outbreaks found that confinement causes a range of psychological effects (Singhal et al., 2024), from fear of spreading disease to severe reactions like impulsiveness, desperation, or even suicide (Alsaleh et al., 2023; Palanichamy Kala et al., 2023). This study defined well-being as mental health, sleep quality, physical health, and psychological resilience. According to the WHO and health models, mental health, which encompasses emotional control, stress response, and psychological function, is a crucial aspect of overall well-being. “Mental health” fits this work’s definition and the broader public health understanding. Ongoing infectious diseases have significantly disrupted global health. This study highlights the urgent need to address these impacts by examining the adverse effects of infectious diseases on well-being as new crises emerge. Disease prevention measures often result in reduced income for low-wage earners, forcing them to stay at home (Palanichamy Kala et al., 2023). Boredom harms psychological health, complicating preventive life (Cohen et al., 2009; Wolkoff et al., 2021; Höckerstedt et al., 2022). Psychological discomfort, frustration, unemployment, and drug abuse often accompany infectious disease outbreaks (Bakshi et al., 2021; Schuster et al., 2022). Restrictions disturb routines and lifestyles (Golding, 2020). Reduced physical activity can increase the risk of welfare issues, such as inactivity and depression (Fruend, 2019). Quarantine also causes fear, financial loss, monotony, and irritation (Shapouri, 2023; Wang et al., 2022). COVID-19, for example, significantly affects physical activity and psychosocial factors (Ganesh et al., 2022; Boentert, 2016; Fruend, 2019).

Extended sitting, lockdowns, and home confinement can negatively affect health and well-being by causing frustration, boredom, and worsening mental health (Boentert, 2016; Fruend, 2019; Robertson et al., 2004; Tomczyk et al., 2022). Isolation increases the risk of cognitive and emotional problems (Boentert, 2016; Chen et al., 2020; Wang et al., 2022). Infectious diseases have heightened these concerns (Al-Jabory et al., 2023; Azak et al., 2023; Schuster et al., 2022), and disrupted routines resulting from limited travel or recreation can be especially challenging for those with personality disorders. The World Health Organization (WHO) recommends steps to curb infectious diseases, including home exercises (Cuijpers et al., 2023; Gronholm et al., 2021). Social isolation is a key response to outbreaks (Clair et al., 2021; WHO, 2023). Physical isolation also affects daily duties. The requirements for physical activity have changed. Infections have had major effects, sometimes conflicting with years of safety advocacy (Clair et al., 2021). Preventive measures impact the well-being of the Chinese population (Gronholm et al., 2021). Despite the interest in health and well-being, research on home-based physical activity (HBPA) and its impact on health is limited. With access to traditional HBPA restricted, it is vital to understand how people adapt to and benefit from HBPA. This research gap presents an opportunity to expand our knowledge and inform public health policies focused on HBPA. This study aimed to assess the mediating role of HBPA in QoL, specifically psychological resilience, physical and mental health, and sleep quality. The following research questions guided this study:

*RQ1:* How does the perceived threat of an infectious disease affect the four dimensions of human well-being: mental health/well-being, sleep quality, physical health, and psychological resilience?
*RQ2:* What is the mediating role of home-based physical activity in the impact of infectious diseases and their preventive measures on human health?

### 1.1 Theoretical framework

Social cognitive theory (SCT) emphasizes the significance of self-efficacy, which refers to an individual’s confidence in their ability to perform behaviors required to achieve specific outcomes (Islam et al., 2023; Riley et al., 2016). Self-efficacy influences home-based physical activity (HBPA). For example, suppose that people believe that they can successfully engage in physical activity at home. They are more likely to participate in these activities, leading to improvements in mental and physical health, sleep quality, and psychological resilience (Schunk and DiBenedetto, 2016; Schunk and DiBenedetto, 2020). Furthermore, emotional characteristics such as psychological resilience (Gürsoy and Toksoy, 2023; McDaniel et al., 2022), physical fitness (Baretta et al., 2019; Islam et al., 2023), and mental health affect individuals’ ability to engage in home-based physical activity (Ertokus, 2022; Frutos et al., 2023; Furushima et al., 2018). Individuals with higher psychological resilience are more likely to engage in physical activity and reap its health benefits (Junge et al., 2013; Noseworthy et al., 2023). The environmental factors represented in the model (infectious diseases and their prevention) can influence behavior. According to SCT, environmental factors such as the availability of resources (e.g., online exercise programs) and social support (e.g., encouragement from family and friends) play a critical role in facilitating or hindering physical activity (Zhang and Zhao, 2023). During an infectious disease outbreak, the constraints of isolation measures can serve as both motivators and barriers to effective action. SCT explains that if the environment provides opportunities for home-based physical activity and individuals perceive support or have access to resources, they are more likely to engage in and maintain physical activity (McNamara et al., 2023). In contrast, physical fitness is positively associated with better health outcomes and a higher quality of life. As individuals engage in daily exercise, their physical fitness improves, which may further enhance their mental well-being (de Rondão et al., 2023; IJntema et al., 2023; Zhang and Zhao, 2023). Adequate sleep is crucial for overall health and well-being, and physical activity improves sleep quality. Therefore, engaging in physical activity may have a positive impact on sleep (Baretta et al., 2019; Compton and Shim, 2020), which may further improve health-related quality of life (WHO, 2023; Yuan et al., 2020). Mental health issues are also crucial aspects of health-related quality of life. Engaging in physical exercise has been shown to positively impact mental health and is associated with numerous other health benefits. In summary, this conceptual framework posits that HBPA may positively impact psychological resilience, physical fitness, sleep, and mental well-being, thereby improving the health-related quality of life of patients with infectious diseases.

### 1.2 Proposed hypotheses operationalization

The emergence of an infectious disease often triggers widespread fear, uncertainty, and stress (Bandura, 2004; Clair et al., 2021; Compton and Shim, 2020). People experience anxiety about contracting the disease, concerns about the well-being of their loved ones, and stress related to societal and economic disruptions caused by the pandemic, such as job losses, isolation, and uncertainty about the future (Clair et al., 2021; Kushwaha et al., 2017). Proposing that infectious diseases negatively impact mental health is crucial because mental well-being is fundamental to overall health. Understanding how a pandemic affects mental health can help inform interventions, such as well-being support services or public health campaigns, to address anxiety, depression, and stress during health crises. Sleep quality is closely linked to both psychological and physical health. Stress and anxiety caused by an infectious disease can lead to sleep disturbances, such as insomnia or restless sleep. People may have trouble sleeping due to worrying about their health, financial insecurity, or disruption to their daily routine. Sleep is essential for the proper functioning of the immune system, mental clarity, and overall health. Understanding how a pandemic affects sleep quality can highlight the need for interventions to promote good sleep hygiene, manage stress, and maintain healthy sleep patterns, even during stressful times. In addition, Azak et al. (2023) explained that the infectious disease problem has also resulted in a significant increase in substance use and abuse as individuals attempt to cope with the stress and uncertainty it caused. Substance abuse can lead to further mind-related problems, including addiction, and exacerbate existing mental health issues (Compton and Shim, 2020; Oftedal et al., 2019). In addition to their physical effects, diseases significantly affect individuals’ mental health, including sleep quality. Noseworthy et al. (2023) proved that the physical health of individuals can be negatively affected during a pandemic in several ways. Reduced physical activity due to lockdowns, sedentary behavior from staying indoors, changes in diet, and disruptions in regular healthcare services can contribute to a decline in health. Additionally, stress and anxiety can have physiological effects, such as increased blood pressure and weakened immune responses. Physical health is a critical component of overall well-being. Understanding the impact of the pandemic on physical health can help inform policies and recommendations for maintaining physical activity and healthy behavior during restrictions. Studies have shown that several infections, such as influenza (flu) and fungal nail infections, have considerably increased sleep problems and disturbances (de Araujo et al., 2022; Jroundi et al., 2015; Polisena et al., 2021; Selleck and Barnard, 2020).

**Figure 1:**
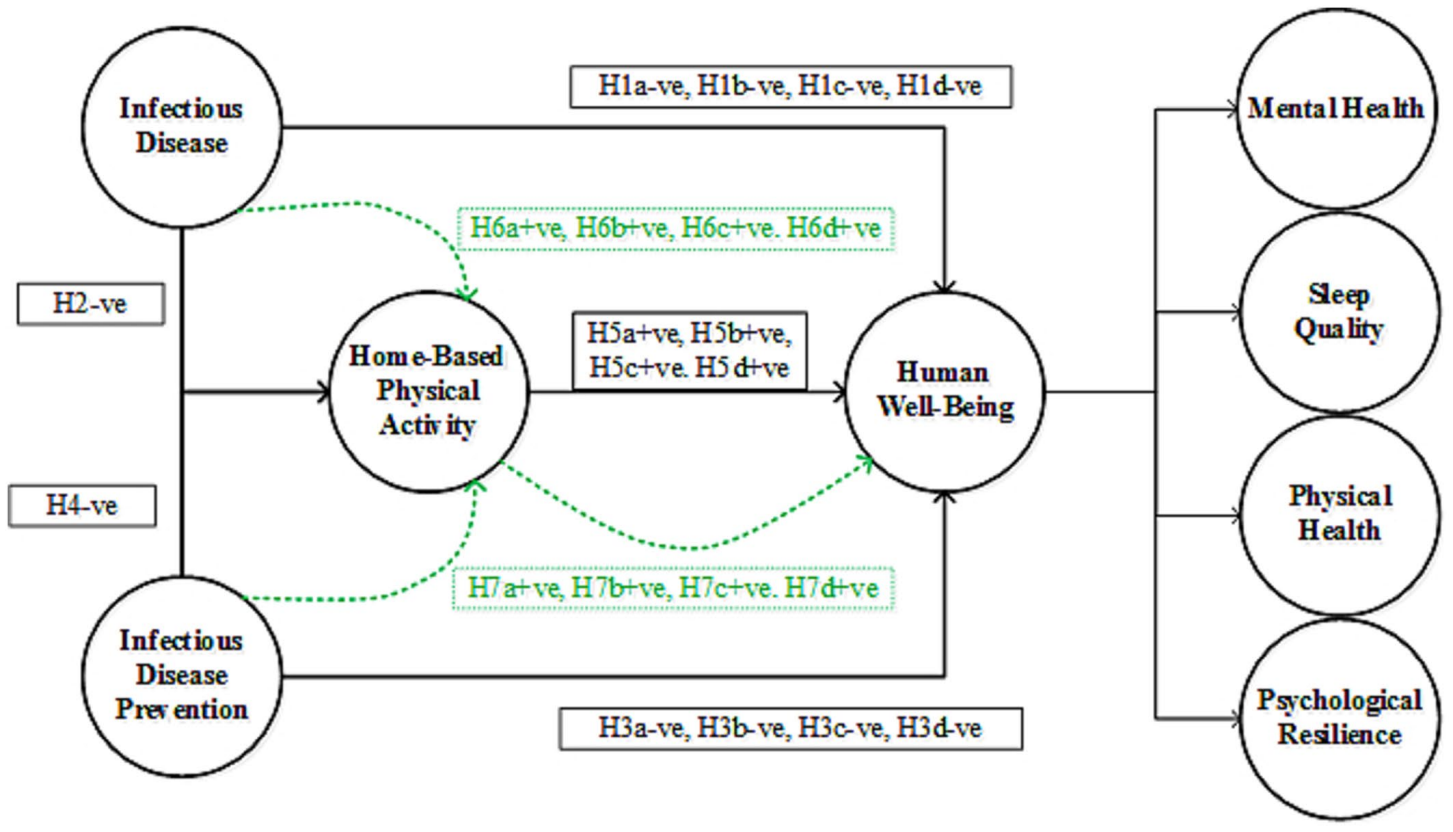
Research framework, where “H” denotes the proposed hypotheses.

Psychological resilience refers to the ability to cope with stress and adversities (Rishipathak et al., 2021). An infectious disease outbreak can test this resilience as people face long-term stressors such as fear of infection, economic hardship, and social isolation (Jroundi et al., 2015; McNamara et al., 2023). Some individuals struggle to adapt, which leads to decreased psychological resilience. Psychological resilience is essential for recovery from trauma and for maintaining mental health during crises. By investigating the negative impact of infectious diseases on resilience, this study highlights the importance of developing interventions that promote resilience, including community support programs, coping strategies and access to mental health resources. Preventive measures, such as staying indoors and limiting physical contact, can reduce opportunities for physical activity, leading to a more sedentary lifestyle (Bandura, 1998; Liu et al., 2020; Weston et al., 2018). This decrease in physical activity can negatively affect cardiovascular health, muscle strength, and overall physical fitness (Baretta et al., 2019; Daryabeygi-Khotbehsara et al., 2021). Disrupted routines may also contribute to unhealthy eating habits and weight gain. Infectious disease prevention measures, such as social distancing, quarantine, and lockdowns, can have significant psychological effects. Being isolated from loved ones, facing restrictions on social interactions, and experiencing disruptions to daily routines can increase feelings of loneliness, anxiety, and depression. Stress and lifestyle changes resulting from preventive measures disrupt the sleep quality. For instance, increased anxiety, lack of routine, and decreased physical activity can lead to sleep disturbances, such as insomnia or irregular sleep patterns (Clement-Carbonell et al., 2021; IJntema et al., 2023). Moreover, concerns regarding the pandemic and the impact of isolation can interfere with sleep. The current literature suggests that infectious diseases have a profound and negative impact on psychological resilience. The following hypotheses are proposed based on the research framework of this study (see Figure 1):

*H1*: Infectious disease has a negative impact on human wellbeing (H1a = mental health, H1b = sleep quality, H1c = physical health, H1d = psychological resilience).

*H2:* Infectious disease has a negative impact on home-based physical activity.

*H3:* Infectious disease prevention has a negative impact on human wellbeing (H3a = mental health, H3b = sleep quality, H3c = physical health, H3d = psychological resilience).

*H4*: Infectious disease prevention has a negative impact on home-based physical activity.

Physical activity has long been recognized as crucial for maintaining overall health and wellness (Anokye et al., 2012; Wu et al., 2022). The relationship between physical exercise and mental health is thought to be mediated by various biological and psychological mechanisms (Hamer et al., 2009; Turner et al., 2019). Infectious diseases and their preventive measures (such as isolation, quarantine, or social distancing) can lead to heightened stress, anxiety, and fear among the general population. Home-based physical activities can serve as an effective tool for reducing these negative psychological effects (Hamer et al., 2009; Weston et al., 2018). For instance, physical activity has been shown to increase endorphin levels (Geraedts et al., 2013), which are known to have mood-enhancing effects. The relationship between physical exercise and physical health is well established (Salerno et al., 2023), with numerous studies demonstrating the positive impact of physical exercise on various health outcomes, including cardiovascular health, weight management, and overall mortality (Kokkinos and Myers, 2010; Salerno et al., 2023; Thomas et al., 2003). Physical exercise is known to release endorphins, the “feel-good” hormones, which help alleviate stress, anxiety, and depressive symptoms, and promote better mental health (Anokye et al., 2012). Simultaneously enhancing physical fitness, boosting mood, and improving sleep promotes a comprehensive sense of well-being. Home-based physical activity is a key behavioral strategy that can improve psychological resilience, physical health, and sleep quality during infectious disease outbreaks. HBPA mediates physical health by improving the functioning of the cardiovascular and musculoskeletal systems (Di Francescomarino et al., 2009). Psychological resilience refers to a person’s ability to cope with stress and adversity and is positively associated with engaging in home-based physical activities (Campbell-Sills et al., 2006; Hamill, 2003). Physical exercise provides a practical and effective means of coping with stressors and physical limitations imposed by health crises, thereby enhancing the overall well-being. Based on the above discussion, the following hypotheses were proposed according to the framework (see Figure 1):

*H5*: Home-based physical activity positively mediates the impact of the infectious disease on human well-being (H5a = mental health, H5b = sleep quality, H5c = physical health, H6d = psychological resilience).

*H6*: Home-based physical activity positively mediates the impact of the infectious disease prevention on human well-being (H6a = mental health, H6b = sleep quality, H6c = physical health, H6d = psychological resilience).

## 2 Methods

### 2.1 Study participants

The two cities in which this study was conducted were Suzhou and Wuhan. Social media was used to recruit participants from a convenience sample of students. Participants had to be at least 20 years old, have completed an undergraduate-level education, and engage in physical activity at home while quarantined to be included in the study. The study adhered to ethical standards, including the Declaration of Helsinki of the World Medical Association, and was approved by the Ethics Committee of Hunan City University (HCU15012025). Informed consent was obtained from all the participants. The administration and design of our survey followed the CHERRIES standards (Eysenbach, 2004).

### 2.2 Data collection tool

The research team developed an initial data collection tool in English based on validated instruments (Fabbri et al., 2021; Lee et al., 2011) and published studies on home-based physical activity and health during isolation (Kokkinos and Myers, 2013; Kokkinos and Myers, 2010). A rational approach was used as a standardized procedure to develop items and refine the multi-item questionnaires. Experts play a critical role in the questionnaire development process (Simms, 2008). This approach is suitable only when the construction of interest is studied briefly. The word “rational” applies to professionals’ ostensibly logical judgments. This technique can also be referred to as a pre-theoretical or an intuitive approach. The rational method for questionnaire development comprises eight steps: theoretical framework, concept analysis, item specification, item production, item judgment, scale construction, validation, and commenting (Simms, 2008). A team of six public health professionals examined the preliminary multi-item questionnaire using a rational method. Two independent translators translated the questionnaire (forward and backward) from the original language to the target language (Tsang et al., 2017). The final questionnaire consisted of 38 items across seven constructs (see Supplementary File): infectious disease (4 items), infectious disease prevention (6 items), home-based physical activities (7 items), mental health (5 items), sleep quality (5 items), physical health (5 items), and psychological resilience (6 items).

### 2.3 Measures

We used a multi-item self-administered questionnaire to evaluate the following study variables: study participants’ demographics, beliefs about infectious diseases, including their impacts and prevention measures, home-based physical activity, mental and physical health, sleep quality, and psychological resilience.

Several measures have been implemented to address the infectious diseases. Travel restrictions inside and between nations, the shutdown of business activities, social distancing and home isolation are some of the examples. Some people find it challenging to determine whether they have been quarantined, isolated, or locked in. Social distancing is detrimental to mental health because it discourages interaction with others (Moreira-Neto et al., 2021). Mental health was assessed in relation to the isolation period and its preventive measures by adapting the “Infectious Diseases, mental health, and wellness survey questionnaire” developed by El Tantawi et al. (2022). The pandemic has significantly affected sleep quality. Sleep quality was assessed using the Chinese version of the Pittsburgh Sleep Quality Index (PSQI) (Mak et al., 2019). Physical health was assessed using self-rated health measures developed by Eriksson et al. (2001). Psychological resilience was evaluated using the Resilience Style Questionnaire (RSQ) developed by Barger (2006). In our study, we used a 5-point Likert scale (1 = strongly disagree, 2 = disagree, 3 = no opinion, 4 = agree, and 5 = strongly agree) to assess COVID-19 and its preventive measures.

Outdoor HBPA is said to be severely curtailed by preventive measures against infectious diseases, which further devastate rapid infections, making it especially important to maintain a safe distance from other people (Geraedts et al., 2013; Salerno et al., 2023). The survey participants were asked to respond on a Likert scale (1 = strongly disagree, 2 = disagree, 3 = no opinion, 4 = agree, and 5 = strongly agree) regarding the role of HBPA in health-related quality of life. As time spent outdoors and access to outdoor spaces have been severely reduced during the pandemic, many people have turned to home-based exercise as a coping mechanism to improve their quality of life. Self-isolation also has a detrimental effect on people’s physical activity levels, and increased screen time adversely affects physical health, happiness, sleep habits, and livability. Consequently, people can practice movements at home by engaging in exercises (Kröönström et al., 2020).

### 2.4 Procedure

Anonymized web-based surveys provided most of the primary data. To ensure a reliable sample size, we conducted a pilot test using a questionnaire with 40 participants in the study. Some questions were revised based on the pilot study results to elicit the most relevant and precise responses. The researchers used WeChat to distribute the link to the final questionnaire in Wuhan and Suzhou, thereby extending its reach beyond the initial point of contact and attracting more participants. Participants were encouraged to share the survey with as many individuals as possible. The questionnaire was completed anonymously using a hyperlink accessible on mobile devices and computers. Only one submission was allowed per IP address, computer, or mobile phone to avoid duplications. In total, 2,300 people were sent a link to the online survey questionnaire along with information about the research and instructions on how to complete and submit it. After a quality check, the answers of 2,200 respondents were considered to compile the results of this study.

### 2.5 Data analysis

The collected data were analyzed using SmartPLS without imposing assumptions on the data distribution, including models with multiple constructs, structural pathways, and indicator variables. Smart PLS is a correlational approach that utilizes a structural equation model, emphasizing forecasting when estimating an empirical relationship with attributes that provide a potential explanation.

## 3 Results

### 3.1 Demographic characteristics of the study participants

The demographics of the 2,200 respondents who completed the online survey, along with their responses, are shown in Table 1. The majority (52.2%) of the survey participants were male, and (47.8%) were female. The age distribution shows that the majority (35.6%) of the survey participants belonged to the 27–33, (21.3%) belonged to the 34–40, (13.6%) belonged to the 41–46, (13.4%) belonged to the 47–53, (9.7%) belonged to the 20–26 and (6.4%) belonged to the 59 + age group. The educational scenario showed that the majority (41.00%) were master’s degree holders, (28.2%) were bachelor’s degree holders, (18.2%) had a graduate level of education, (8.3%) were associate’s degree holders, (2.8%) were undergraduates, and (1.5%) were in other categories. Occupation statistics showed that (32.9%) were private employees, (29.7%) were self-employed, (22.3%) were government employees, (10.9%) were students, (0.9%) were retired, (0.8%) had no occupation, and (2.5%) were in other occupations. Most (80.5%) of the survey participants were married, (18.4%) were unmarried, and (1.1%) were otherwise.

**Table 1:** Survey participants’ demographics (n-2200).

Variables	Categorization	Frequency/Percentage	Mean	SD
Gender	Male	1,148 (52.2%)	1.33	0.469
	Female	1,052 (47.8%)	1.33	0.469
Age	20–26	213 (9.7%)	1.44	0.613
	27–33	783 (35.6%)	1.44	0.613
	34–40	469 (21.3%)	1.44	0.613
	41–46	300 (13.6%)	1.44	0.613
	47–53	294 (13.4%)	1.44	0.613
	53 +	141 (6.4%)	1.44	0.613
Education	Undergraduate	62 (2.8%)	1.93	0.461
	Associate Degree	183 (8.3%)	1.93	0.461
	Bachelor Degree	620 (28.2%)	1.93	0.461
	Master Degree	901 (41.0%)	1.93	0.461
	Graduate	400 (18.2%)	1.93	0.461
	Others	34 (1.5%)	1.93	0.461
Occupation	Government Employee	491 (22.3%)	2.82	0.864
	Private Employee	724 (32.9%)	2.82	0.864
	Self-employed	653 (29.7%)	2.82	0.864
	Retired	19 (0.9%)	2.82	0.864
	Student	240 (10.9%)	2.82	0.864
	No occupation	17 (0.8%)	2.82	0.864
	Others	56 (2.5%)	2.82	0.864
Marital status	Married	1770 (80.5%)	1.79	0.407
	Unmarried (single)	405 (18.4%)	1.79	0.407
	Others (divorced, widowed)	25 (1.1%)	1.79	0.407

### 3.2 Convergent validity

The internal consistency of the measurements was evaluated using Cronbach’s alpha (*α*) and composite reliability (CR). It varied from (0.823 to 0.943) and (0.828 to 0.945), respectively, both above the 0.70 cut-off limit (Klein, 2016). Convergent validity testing was performed for each factor loading, and the average variance was determined. The average variance extracted (AVE) in statistics (classical test theory) measures the proportion of variation captured by a construct to the variation resulting from the estimation errors. All items had loadings of > 0.6. The AVE for all variables was above the 0.5 threshold, showing convergent validity (see Table 2).

**Table 2:** Reliability and validity results.

Items	Loadings	α	Cr	AVE
Infectious disease		0.863	0.864	0.709
ID 1	0.829			
ID 2	0.862			
ID 3	0.847			
ID 4	0.829			
Home-based physical activity		0.869	0.871	0.561
HBPA 1	0.687			
HBPA 2	0.736			
HBPA 3	0.670			
HBPA 4	0.777			
HBPA 5	0.779			
HBPA 6	0.824			
HBPA 7	0.757			
Infectious disease preventions		0.891	0.904	0.647
IDPs1	0.740			
IDPs2	0.771			
ODPs3	0.808			
IDPs4	0.837			
IDPs5	0.841			
IDPs6	0.824			
Mental health		0.823	0.828	0.586
MH 1	0.752			
MH 2	0.807			
MH 3	0.818			
MH 4	0.741			
MH 5	0.703			
Sleep quality		0.849	0.856	0.626
SQ 1	0.769			
SQ 2	0.836			
SQ 3	0.834			
SQ 4	0.809			
SQ 5	0.698			
Physical health		0.867	0.871	0.652
PhysH1	0.793			
PhysH2	0.832			
PhysH3	0.758			
PhysH4	0.849			
PhysH5	0.803			
Psychological resilience		0.943	0.945	0.778
PsyR1	0.868			
PsyR2	0.880			
PsyR3	0.906			
PsyR4	0.859			
PsyR5	0.885			
PsyR6	0.894			

ID, infectious disease; HBPA, home-based physical activity; IDPs, infectious disease preventions; MH, mental health; SQ, sleep quality; PhyH, physical health; PsyR, psychological resilience.

### 3.3 Discriminant validity

Two approaches, heterotrait-monotrait (HTMT) and Fornell–Larcker, were used to assess discriminant validity (Campbell and Fiske, 1959). The correlation between the two constructs must be smaller than the square root of the average variance recovered by the measure. All the estimated average variance square roots were more significant than their associated inter-correlation values (Table 3).

**Table 3:** Discriminant validity.

**Constructs**	**Fornell-Larcker Criterion**	**Fornell-Larcker Criterion**	**Fornell-Larcker Criterion**	**Fornell-Larcker Criterion**	**Fornell-Larcker Criterion**	**Fornell-Larcker Criterion**	**Fornell-Larcker Criterion**
	**HBPA**	**ID**	**IDPs**	**MH**	**PhyH**	**PsyR**	**SQ**
HBPA	0.749						
ID	−0.444	0.842					
IDPs	−0.365	0.71	0.804				
MH	0.475	−0.523	−0.493	0.766			
PhyH	0.553	−0.522	−0.472	0.587	0.808		
PsyR	−0.028	−0.052	0.108	−0.112	−0.092	0.882	
SQ	0.496	−0.392	−0.387	0.548	0.685	−0.126	0.791
Constructs	Heterotrait-Monotrait Ratio (HTMT)	Heterotrait-Monotrait Ratio (HTMT)	Heterotrait-Monotrait Ratio (HTMT)	Heterotrait-Monotrait Ratio (HTMT)	Heterotrait-Monotrait Ratio (HTMT)	Heterotrait-Monotrait Ratio (HTMT)	Heterotrait-Monotrait Ratio (HTMT)
HBPA	1						
ID	0.513	1					
IDPs	0.408	0.797	1				
MH	0.556	0.614	0.555	1			
PhyH	0.626	0.602	0.526	0.701	1		
PsyR	0.059	0.065	0.117	0.13	0.104	1	
SQ	0.565	0.455	0.433	0.655	0.802	0.144	1

ID, infectious disease; HBPA, home-based physical activity; IDPs, infectious disease preventions; MH, mental health; SQ, sleep quality; PhyH, physical health; PsyR, psychological resilience.

### 3.4 Structural equation modelling

During the SEM analysis, model fitness was assessed using the SRMR, d_ULS, d_G, Chi-square, and NFI values. The calculated model fitness values are presented in Table 4 and meet the requirements for SEM analysis.

**Table 4:** Model fitness results.

Indices	Indices	Saturated model	Results
Standardized root mean square residual	SRMR	0.058	< 0.08, good model fit
Squared euclidean Distance (unweighted least squares)**	d_ULS	2.503	indicate a better fit;
Geodesic distance	d_G	0.74	indicate a better fit
chi-square discrepancy	Chi-square	9786.338	In PLS-SEM, chi-square is reported but not used for model acceptance, because PLS is variance-based, not covariance-based
Normed fit index	NFI	0.821	Values > 0.80 indicate acceptable fit; >0.90 is good

**Indicates level of significance.

**Figure 2:**
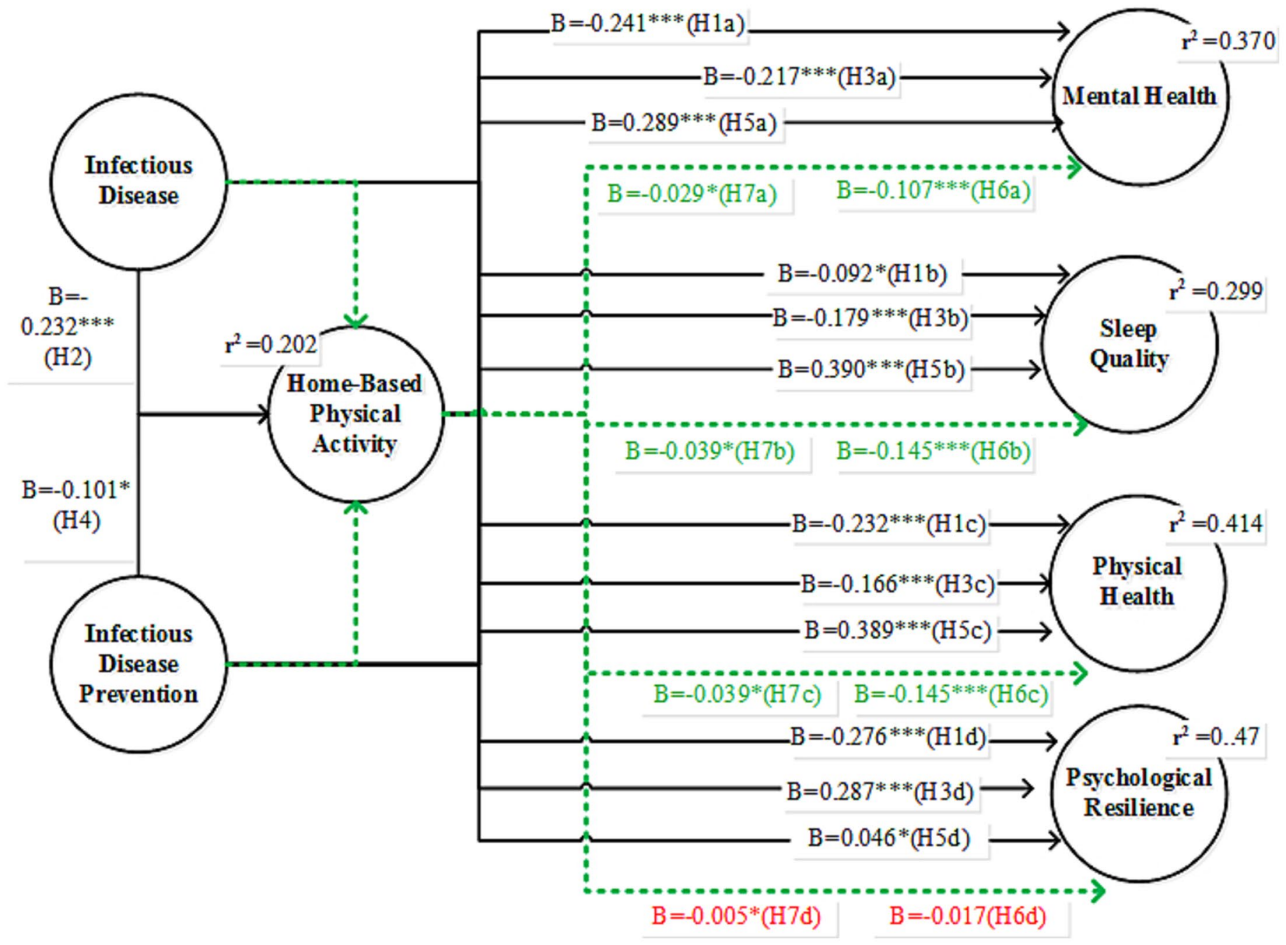
SEM results, where green values present mediating effects and red values are insignificant.

Path coefficient, coefficient of determination for endogenous variables, effect size, multicollinearity, and predictive relevance were calculated to evaluate the models. Table 5 and Figure 2 present the results of the hypothesis testing. First, the relationship between infectious diseases and mental health was negative and significant (*β* = −0.241, *t* = 7.215). This suggests that infectious diseases have strong adverse effects on mental health, contributing to increased stress, anxiety, and other psychological challenges in patients. This hypothesis is supported by the findings of this study, which confirm that mental health deteriorates significantly during an infectious disease outbreak. The impact of infectious diseases on sleep quality was also negative and significant, although less pronounced (*β* = −0.092, *t* = 2.437). This indicates that the pandemic disrupted individuals’ sleep patterns, potentially due to anxiety, fear, and uncertainty. This hypothesis is supported by the adverse effects of infectious diseases on sleep quality. The relationship between infectious diseases and physical health was negative and significant (*β* = −0.232, *t* = 6.265). This shows that the pandemic led to a decline in physical health, possibly due to reduced mobility, restricted access to fitness facilities, or a more sedentary lifestyle. Thus, this hypothesis was supported. Infectious diseases negatively affected psychological resilience (*β* = −0.276, *t* = 9.354), indicating a significant reduction in individuals’ ability to cope with stress and adversity during the pandemic. This hypothesis was supported, emphasizing the harmful effects of infectious diseases on the psychological resilience. Finally, the relationship between infectious diseases and home-based physical activity (HBPA) was negative and significant (*β* = −0.372, *t* = 9.131). This suggests that the pandemic limited individuals’ ability or motivation to engage in physical activity at home, likely because of stress, a lack of routine, or limited space. Thus, this hypothesis is supported.

**Table 5:** SEM results.

Hypotheses	Coefficients	SD-Values	T-Values		Decision	
Direct path						
ID → MH (H1a)	−0.241***	0.033	7.215		Supported	
ID → SQ(H1b)	−0.092*	0.038	2.437		Supported	
ID → PhyH(H1c)	−0.232***	0.037	6.265		Supported	
ID → PsyR(H1d)	−0.276***	0.030	9.354		Supported	
ID → HBPA(H2)	−0.372***	0.041	9.131		Supported	
IDPs → MH(H3a)	−0.217***	0.036	6.053		Supported	
IDPs → SQ(H3b)	−0.179***	0.033	5.375		Supported	
IDPs → PhyH(H3c)	−0.166***	0.034	4.855		Supported	
IDPs → PsyR(H3d)	0.287***	0.028	10.321		Supported	
IDPs → HBPA(H4)	−0.101*	0.041	2.478		Supported	
HBPA → MH(H5a)	0.289***	0.026	11.065		Supported	
HBPA → SQ(H5b)	0.390***	0.030	13.014		Supported	
HBPA → PhyH(H5c)	0.389***	0.029	13.540		Supported	
HBPA → PsyR(H5d)	−0.046*	0.023	2.006		Supported	
Indirect path						
ID → HBPA → MH(H6a)	−0.107***	0.016	6.834	−0.139	−0.077	Supported
ID → HBPA → SQ(H6b)	−0.145***	0.020	7.209	−0.188	−0.108	Supported
ID → HBPA → PhyH(H6c)	−0.145***	0.019	7.471	−0.185	−0.109	Supported
ID → HBPA → PsyR(H6d)	0.017	0.009	1.888	0.001	0.006	Not-Supported
IDPs → HBPA → MH(H7a)	−0.029*	0.012	2.355	−0.054	−0.016	Supported
IDPs → HBPA → SQ(H7b)	−0.039*	0.016	2.418	−0.072	−0.018	Supported
IDPs → HBPA → PhyH(H7c)	−0.039*	0.016	2.388	−0.073	−0.018	Supported
IDPs → HBPA → PsyR(H7d)	0.005	0.003	1.475	0.000	−0.001	Not-Supported

ID, infectious disease; HBPA, home-based physical activity; IDPs, infectious disease preventions; MH, mental health; SQ, sleep quality; PhyH, physical health; PsyR, psychological resilience.***,*Indicates level of significance.

Infectious disease prevention measures (IDPs) had a significant negative effect on mental health (*β* = −0.217, *t* = 6.053). This suggests that strategies such as social distancing and quarantine may negatively impact mental well-being, possibly due to isolation or limited social interaction. Thus, this hypothesis is supported. The effect of IDPs on sleep quality was negative and significant (*β* = −0.179, *t* = 5.375). Preventive measures may lead to sleep disturbances, potentially caused by disrupted routines or anxiety regarding ongoing health crises. Thus, this hypothesis is supported. IDPs had a significant negative impact on their physical health (*β* = −0.166, *t* = 4.855), indicating that restrictions, such as staying indoors, contributed to physical inactivity or deterioration in health. Thus, H3c is supported.

Interestingly, IDPs had a positive effect on psychological resilience (*β* = 0.287, *t* = 10.321). This finding suggests that while preventive measures can create stress, they also promote adaptive coping mechanisms, possibly through the development of new routines or support systems. The relationship between IDPs and HBPA was negative but significant (*β* = −0.101, *t* = 2.478). This suggests that preventive measures slightly reduced the likelihood of engaging in physical activity at home, possibly due to a lack of motivation and/or stress. Thus, this hypothesis is supported.

The mediating role of home-based physical activity (HBPA) was evaluated using a bootstrapping procedure with 5,000 subsamples and bias-corrected 95% confidence intervals. Home-based physical activity positively affected mental health (*β* = 0.289, *t* = 11.065). Engaging in exercise can help alleviate stress and improve mood, ultimately leading to enhanced mental well-being. In H5b, HBPA significantly improved sleep quality (*β* = 0.390, *t* = 13.014), indicating that regular physical activity at home contributes to better sleep, possibly by reducing stress and improving fatigue. The impact of HBPA on physical health was highly positive (*β* = 0.389, *t* = 13.540). Home-based exercise helps maintain or enhance physical fitness and supports overall health (H5c). The relationship between HBPA and psychological resilience was negative but significant (*β* = −0.046, *t* = 2.006). This result was unexpected and suggests that, although small, some aspects of physical activity may not directly contribute to building psychological resilience. Thus, Hypothesis (H5d) is supported. Furthermore, the indirect effects of HBPA indicated that the indirect effect of infectious diseases on mental health through HBPA was negative and significant (*β* = −0.107, *t* = 6.834, 95% CI [−0.139, −0.077]). This suggests that the decline in physical activity due to the pandemic partially mediated its impact on mental health. The indirect effect on physical health was negative and significant (*β* = −0.145, *t* = 7.471, 95% CI [−0.183, −0.107]), indicating that lower HBPA levels during the pandemic contributed to the decline in physical health. The indirect effect of psychological resilience was not significant (*β* = 0.017, *t* = 1.888, 95% CI [0.001, 0.036]). This finding indicates that HBPA does not mediate the relationship between infectious diseases and resilience. The indirect effect of IDPs on mental health through HBPA was negative and significant (*β* = −0.029, *t* = 2.355). This finding implies that preventive measures can reduce HBPA levels and negatively impact mental health. The indirect effect on sleep quality was negative and significant (*β* = −0.039, *t* = 2.418), indicating that reduced HBPA due to preventive measures was associated with poor sleep quality. Thus, this hypothesis is supported. The indirect effect on physical health was negative and significant (*β* = −0.039, *t* = 2.388), indicating that lower HBPA resulting from preventive measures contributed to declining physical health. Finally, the indirect effect on psychological resilience was not significant (*β* = 0.005, *t* = 1.475), suggesting that HBPA did not mediate the relationship between preventive measures and resilience.

## 4 Discussion

This study investigated how perceptions of infectious diseases (ID) and preventive behaviors (IDPs) influence multiple dimensions of well-being and whether home-based physical activity (HBPA) serves as a mediating mechanism. Infectious diseases have been labeled a global pandemic by the World Health Organization (WHO), which has recommended numerous preventive measures to combat them (Mahase, 2020). Different infections significantly affected outdoor physical activity and psychological resilience of the participants. The psychological consequences of large-scale outbreaks of rapidly spreading viral infections can shed light on their effects on mental health. Anxiety, depression, panic attacks, cognitive symptoms, and delirium are common psychiatric symptoms of infectious diseases, such as SARS and MERS. Anxiety, irritability, depression, memory loss, tiredness, posttraumatic stress disorder, and sleeplessness are typical symptoms of these conditions. Many challenges in the early stages of COVID-19, such as infection, financial loss, and quarantine, have led to psychological health problems such as anxiety and depression. This forecast was made with the worldwide dominance of anxiety and sadness peaking at 28 and 26%, respectively. Infectious illness outbreaks have been shown to affect the entire public, including healthcare personnel.

The results indicate that the perceived threat of infectious diseases significantly impacts human well-being. Specifically, the findings showed that infectious diseases negatively affected mental health (*β* = −0.241, *t* = 7.215), sleep quality (*β* = −0.092, *t* = 2.437), and physical health (*β* = −0.232, *t* = 6.265), indicating that stress, anxiety, and disrupted routines contributed to poorer overall well-being. Individuals who perceived higher levels of infectious disease threats reported lower mental well-being, poorer sleep quality, reduced physical health, and lower psychological resilience. Current outcomes for human well-being highlight the psychological burden associated with disease-related uncertainty, fear of infection, and disruption of daily routines during the pandemic. Additionally, the impact on psychological resilience was negative and significant (*β* = −0.276, *t* = 9.354), suggesting that the persistent stress and fear associated with the pandemic reduced individuals’ ability to cope effectively. A decline in psychological resilience indicates that prolonged exposure to stress and disease-related concerns weakens individuals’ adaptive capacity. Thus, RQ1 was answered by demonstrating that infectious diseases substantially worsen mental, physical, and sleep-related well-being and compromise the resilience. Collectively, these results underscore the multifaceted influence of infectious disease threats on overall well-being. However, the current study revealed that both infectious diseases and preventive measures significantly affect various aspects of human well-being, including mental health, sleep quality, and physical health status. However, psychological resilience appeared to be less affected by these factors than other variables. Individuals in many countries may react emotionally, cognitively, physically, or behaviorally to the pandemic. The present pandemic has been associated with several detrimental mental health effects, including sadness, anxiety, post-traumatic stress disorder symptoms, insomnia, and even suicidal ideation. Fear-related behaviors, such as increased distress and mental disorder rates in the community, further contributed to an increase in indirect mortality during the Ebola epidemic in West Africa. Anxiety, worry, despair, and social isolation have all been reported to increase with a general decline in the respondents’ sense of well-being. In contrast, the indirect path results show that safety measures also impact health. Most people are subjected to unexpected circumstances of uncertain duration during confinement, which leads to anxiety, dread, despair, and sleep disturbances. Suicide attempts are made because of a combination of factors, including lack of social interaction, poor mental hygiene practices, and exposure to pessimistic information and media. Being home alone, unable to move about, and unable to see friends and family also affected their well-being negatively. Similar to how people think about becoming sick, taking steps to avoid getting sick negatively affects their mental, sleep, and physical health. Current effects may be caused by being locked up for a long time, not being able to contact others, lifestyle changes, and not knowing what will happen next. Preventive activities enhance psychological resilience rather than detrimental connections. In limited environments, individuals may actively employ coping strategies, adapt, and utilize their personal resources. The dual impact of the pandemic revealed that preventive actions can be complex for both the mind and body, but they can also help people become stronger psychologically.

Home-based physical activity (HBPA) has emerged as a significant mediator in several pathways. HBPA significantly mediated the relationship between infectious diseases and several aspects of well-being. The indirect effects revealed that HBPA positively influenced mental health (*β* = −0.107, *t* = 6.834), sleep quality (*β* = −0.145, *t* = 7.209), and physical health (*β* = −0.145, *t* = 7.471), suggesting that engaging in physical activity helps buffer the negative effects of the disease on these well-being outcomes. Higher engagement in physical activity contributes to improved mental well-being, better sleep quality, and enhanced physical health. The current findings align with established evidence that physical activity reduces stress, improves mood, regulates sleep cycles, and supports physiological functioning. Mediation analyses further revealed that reduced physical activity partially explained the adverse effects of perceptions of infectious diseases and preventive measures on these well-being outcomes. Thus, promoting physical activity during periods of restricted movement may buffer the detrimental effects of disease-related stressors and lifestyle limitations on mental health of patients with DM. However, HBPA did not mediate the relationship between infectious diseases and psychological resilience, as this effect was not statistically significant (*β* = 0.017, *t* = 1.888). This indicates that while HBPA effectively enhances mental, sleep, and physical health, it may not be sufficient on its own to improve resilience, which may require additional psychological or social intervention. Therefore, RQ2 was partially answered, showing that HBPA mediates some, but not all, aspects of well-being. Moreover, the inverse relationship between home-based physical activity and psychological resilience, although statistically significant, was small and unexpected in this study. This result may reflect variations in individuals’ psychological responses to physical activity during periods of stress. For example, less resilient individuals may intentionally increase their physical activity as a coping mechanism, which could result in a negative correlation between physical activity and resilience.

Interestingly, while infectious diseases negatively affected psychological resilience (H1d: *β* = −0.276), preventive measures showed a positive effect (H3d: *β* = 0.287). This may indicate that some individuals develop adaptive coping strategies in response to structured measures, such as finding new routines or engaging in supportive activities. Isolation from home may have lasting adverse effects on mental and physical health. Home confinement in China exacerbated mental health issues, including anger, boredom, and isolation, as well as mood disorders such as despair, stress, and anxiety. The negative impact on sleep quality (H1b and H3b) suggests that the disease and preventive measures disrupted sleep patterns, which is consistent with studies showing increased sleep disturbances during the pandemic. This may be due to heightened anxiety and alterations in daily routines. An individual’s physical and mental health may be affected by the social and psychological effects of living alone. Sadness, cognitive decline, and unease result from prolonged solitude. However, our results also demonstrated a decrease in physical health (H1c and H3c) due to reduced mobility and a more sedentary lifestyle during the lockdown, findings that are supported by previous studies. This highlights the physical health risks associated with prolonged isolation. Government restrictions on travel and outdoor activities disrupted the everyday routines and lifestyles of the Chinese people. According to a Chinese study, (53.8%) of the Chinese public experienced severe to moderately unfavorable psychological resilience effects owing to infections (Schunk and DiBenedetto, 2020; Selleck and Barnard, 2020). Meditation, an ancient form of exercise that focuses on both the mind and body, has been particularly beneficial in improving psychological well-being through emotional adjustment and neurogenic stress-coping behaviors, suggesting that almost all types of yoga can help reduce stress. Exergames, also known as fitness video games, are gaining popularity among young people as a home hobby. Aerobic exercises, such as dancing, Pilates-style balance and flexibility training, weightlifting, and other strength and endurance exercises, can all be performed in the comfort of one’s own home. According to their study, people who increased their leisure-related physical activity (walking and moderate physical activity) reported better physical health. The cognitive benefits of regular exercise vary according to the frequency of exercise. People who exercised regularly throughout the pandemic felt more emotional.

## 5 Conclusion

HBPA has been shown to positively affect self-perception, emotional regulation, and quality of life of those living in the pandemic. Post-traumatic stress disorder (PTSD) may respond to home fitness programs that use virtual reality. Our research shows that HBPA improves the quality of life during the pandemic. HBPA enhances receptivity to anxiety-related cues and lowers anxiety sensitivity, both of which are beneficial for mental health. As the duration of the pandemic lockdown increased, light physical activities such as strolling and non-traditional forms of exercise, including meditation and creative pursuits, became popular. Appropriate psychological care guidelines for the general population and healthcare workers combating epidemics should be developed. All these strategies would aid in maintaining optimal infectious disease patient care and preventing adverse mental health outcomes, particularly suicides.

The current study has some limitations that should be considered in future studies. First, convenience sampling was used to collect data from adults in two Chinese cities, which may limit the generalizability of the findings to other areas, cultures, and demographic groups. More diverse and representative samples from different regions are required for future studies. Second, the study examined attitudes toward respiratory infectious diseases and their prevention; therefore, its conclusions cannot be applied to vector-borne, waterborne, or systemic infections. Future research should study how viral diseases affect psychological and behavioral responses. Third, the cross-sectional design limited the ability to make causal inferences. Although SEM mediation patterns provide a theoretical direction, longitudinal or experimental research is necessary to demonstrate the temporal sequences and causal relationships between variables. Ultimately, psychological resilience is influenced by the complex interplay of psychosocial and environmental factors. Because this study did not capture variables such as social support, coping strategies, or personality characteristics, the resilience findings should be interpreted with caution.

## Data Availability

The data analyzed in this study is subject to the following licenses/restrictions: dataset belong to an ongoing project and available on request to corresponding author. Requests to access these datasets should be directed to Rashid Menhas, menhas.r@yahoo.com.
