# Supplementary material for: Home-based physical activity and well-being during infectious diseases: a structural equation modeling study
Source: Front Psychol. 2026 Jan 13;16:1732564. doi: 10.3389/fpsyg.2025.1732564 (PMC12834825; doi:10.3389/fpsyg.2025.1732564)
Supplement: Supplementary file 1 [file Table_1.docx]

**Construct and Scale Items**

| **Construct/**  **Variables** | **Items/domain (5 Points Likert Scale)** |
| --- | --- |
| Infectious Disease (ID) | 1. The infectious disease affected general health 2. The infectious disease affected the ability of your household 3. The infectious disease created xenophobia 4. The infectious disease affected the physical and psychological quality of life |
| Infectious Disease Prevention (IDPs) | 1. Frequent body examination 2. Vaccination for infection 3. Social distance 4. Wearing a mask 5. Avoid going to risky human 6. Hand washing, active living, and personal hygiene |
| Home-based Physical Activity (HBPA) | 1. Virtual reality fitness, app-based exercise, yoga, dancing, boxing, running, walking, and jogging promote active living, which is helpful in overall psychological resilience 2. Quality of life improved due to home-based physical activity during the infectious disease 3. Home-based physical activity during infectious disease is helpful for the improvement of psychological resilience 4. Home-based physical activity during an infectious disease is helpful for the improvement of physical health 5. Home-based physical activity during the novel infectious disease is helpful for the improvement of mental health 6. Home-based physical activity during an infectious disease helps improve sleep quality 7. Home-based physical activity during the infectious disease is helpful for the improvement of natural resistance against the novel Coronavirus disease |
| Mental Health (MH) | 1. The infectious disease created fear and anxiety 2. The infectious disease created mental stress 3. The infectious disease created mental depression 4. The infectious disease created bipolar disorder 5. Substance abuse or addiction during the isolation was used as a source of comfort |
| Sleep Quality (SQ) | 1. The infectious disease has impacted the regular sleep of 8 hours 2. The infectious disease has an impact on your sleep 3. During an infectious disease, one has an insomnia problem 4. The sleep quality was affected due to the hopelessness and suicidal thoughts resulting from the infectious disease 5. Started to use the substance for sleep or to improve the sleep quality |
| Physical Health (PhyH) | 1. During the infectious disease, my physical health was not affected 2. During the infectious disease, my physical health was affected due to sedentary behavior 3. Negative psychological factors (anxiety, depression, stress, fears) have impacted physical health 4. Physical health improved by practicing home-based physical activities during the infectious disease home isolation 5. The infectious disease measures affected physical health negatively |
| Psychological Resilience  (PsyR) | 1. My worries about the infectious disease overwhelmed me 2. I have deep inner strength during the infectious disease 3. The infectious disease impacted emotions negatively 4. The infectious disease impacted life satisfaction and self-compassion 5. My attitude and behavior remained positive toward life 6. Psychological impacts of the infectious disease impacted the overall psychological resilience |
